# College and Hospital Notes

**Published:** 1906-06

**Authors:** 


### COLLEGE AND HOSPITAL NOTES.

The new Buffalo Municipal Hospital is to be completed, according to a recent announcement, and turned over to the health department early in June. It is erected on the site of the old "pest-house" in East Ferry Street, and cost about $50,000. This will enable the department to give appropriate care to smallpox patients and those affected with other contagious diseases. It is probable that a portion of the building will be used as a detention hospital for suspected smallpox cases.

---

The Pocahontas Hospital will be completed first of all the buildings at the Jamestown Exposition. The wisdom of a properly constructed and equipped hospital with a competent medical director and an efficient staff of assistants and nurses, was the lesson taught by experience at the Pan-American Exposition of 1901, and it has been accentuated by later expositions.

The Pocahontas hospital has offices for the medical staff, sleeping apartments for house doctors, nurses, and the necessary help.

There are bright, airy wards, both for male and female patients, and private rooms for such sufferers as may be too ill to occupy cots in the wards and require isolation. In the basement will be a kitchen, dining room, store-rooms, and apart from these there will be installed a first-class heating outfit.

The hospital will contain a room for medical and surgical supplies, a sterilising room, and an operating theater, planned and equipped according to the most modern methods. A complete outfit of hospital instruments and appliances will be placed in this building.

Already a competent corps of resident surgeons and nurses is being congregated. These will live in the hospital, and be ready at any hour of the day or night to care for emergency cases needing attention.

---

The Alumni Association of the Medical Department of the University of Buffalo has appointed its thirty-first annual meeting for May 29-June 1, 1906. Some of our readers will receive this issue of the Journal in time to be interested in this abstract of the program, taken from the circular of the Executive Committee.

In accordance with the resolution passed last year, the Executive Committee has arranged for a four-day meeting this year to be held during Tuesday, May 29, Wednesday, May 30, Thursday, May 31, and Friday, June 1, 1906.

Our Alumni who have medical and surgical services at the following institutions, have signified their willingness to present cases of interest.

Buffalo General Hospital, Hospital of the Sisters of Charity, Erie County Hospital, State Hospital for the Insane, Children's Hospital, German Hospital, Emergency Hospital, Providence Retreat, German Deaconess's Hospital, Woman's Hospital, Riverside Hospital, Eye and Ear Infirmary, Charity Eye, Ear and Throat Infirmary, Dispensary of the German Hospital, Dispensary of the Emergency Hospital, Dispensary of the University of Buffalo.

The Alumni are urged to present cases of interest for discussion, diagnosis or treatment. To facilitate the arrangements, alumni are requested to advise, if possible, the chairman of the executive committee as to the nature of the case and at whose clinic it is desired to present it. Detailed schedule of clinics with hours and clinical lecturer will be presented later..

The classes of '56, '66, '76, '86 and '96 will meet in reunion.

All members of the association are requested to register as soon as possible after arriving in town. The registry desk will be in the main hall of the college building, where the detailed program of the four days may be found; members of the reception committee will gladly answer any questions relating to the various clinics and other features of the program.

The Executive Committee again urges all alumni to enroll themselves as active members of the association. The advancement and success of the University is a benefit to each alumnus and he should show his loyalty to his Alma Mater by furthering the good work by his attendance, efforts and contributions.

The Executive Committee wishes to announce that it has been so fortunate as to secure the consent of Dr. Denslow Lewis, of Chicago, to give an address on Thursday evening upon the great question of the day, "The Social Evil." Dr. Lewis's international reputation as well as his special knowledge upon this topic need no repetition. His acceptance assures the association a rare treat.

---

THE Saint Louis University held the commencement of its medical department,—the Marion Sims-Beaumont College of Medicine,—at the Odeon, May 19, 1906. The JOURNAL acknowledges the receipt of an invitation to attend the exercises, through an undergraduate, Mr. Edward Lee Dorsett, son of Dr. Walter Blackburn Dorsett of Saint Louis. We very much regret that we were unable to be present on that occasion.

---

THE Medical College of Virginia held its sixty-eighth annual commencement at the Academy of Music, Richmond, May 15, 1906, at 8.30 p. m. The eighteenth annual meeting of the Society of the Alumni was held during May 13, 14, and 15, which latter included a baccalaureate sermon on Sunday evening, May 13, clinics, business meeting, and smoker during Monday, 14th and clinics, regular meeting of the Alumni, and commencement exercises during Tuesday, 15th; also a reception by the faculty at Masonic Temple Tuesday evening. The JOURNAL takes this method of acknowledging the receipt of an invitation to all these ceremonies which we were unable to attend.

---

THE Lexington Heights Hospital Training School held its commencement exercises at the First Congregational Church, Buffalo, Thursday Evening, May 17, 1906, nine nurses being graduated. Music, flowers, and an address by Mr. Robert G. Freeman, served to make an interesting evening. After the exercises at the church were concluded, Dr. and Mrs. DeWitt G. Wilcox, of West Ferry Street, gave a reception at their home in honor of the graduates, which was attended by more than 300 guests.

